# Mindin Activates Autophagy for Lipid Utilization and Facilitates White Spot Syndrome Virus Infection in Shrimp

**DOI:** 10.1128/mbio.02919-22

**Published:** 2023-02-13

**Authors:** Jie Gao, Ye-Jia Song, Hao Wang, Bao-Rui Zhao, Xian-Wei Wang

**Affiliations:** a Shandong Provincial Key Laboratory of Animal Cells and Developmental Biology, School of Life Sciences, Shandong University, Qingdao, Shandong, China; b State Key Laboratory of Microbial Technology, Shandong University, Qingdao, Shandong, China; c Laboratory for Marine Biology and Biotechnology, Qingdao National Laboratory for Marine Science and Technology, Qingdao, Shandong, China; Uppsala University; University of Pennsylvania

**Keywords:** white spot syndrome virus, shrimp, Mindin, autophagy, lipid droplet

## Abstract

Mindin is a secreted extracellular matrix protein that is involved in regulating cellular events through interacting with integrin. Studies have demonstrated its role in host immunity, including phagocytosis, cell migration, and cytokine production. However, the function of Mindin in the host-virus interaction is largely unknown. In the present study, we report that Mindin facilitates virus infection by activating lipid utilization in an arthropod, kuruma shrimp (Marsupenaeus japonicus). Shrimp Mindin facilitates white spot syndrome virus infection by facilitating viral entry and replication. By activating autophagy, Mindin induces lipid droplet consumption, the hydrolysis of triglycerides into free fatty acids, and ATP production, ultimately providing energy for virus infection. Moreover, integrin is essential for Mindin-mediated autophagy and lipid utilization. Therefore, by revealing the mechanism by which Mindin facilitates virus infection through regulating lipid metabolism, the present study reveals the significance of Mindin in the host-virus interaction.

## INTRODUCTION

Mindin, also known as spondin 2 or M-spondin, is a secreted extracellular matrix (ECM) protein. Consisting of an N-terminal spondin domain and a C-terminal thrombospondin type 1 repeat (TSP), this protein is structurally conserved from *Drosophila* to zebrafish and mammals (1–3). As an ECM protein, Mindin is involved in the interaction between cells and their environment. Studies have demonstrated the significance of Mindin, using a mouse model, in host immune responses, including phagocytosis, cell migration, and cytokine production (4–8). Mindin-deficient mice showed resistance to lipopolysaccharide-induced septic shock and impaired clearance of invading bacteria. Using recombinant protein, Mindin was found to directly bind bacteria and bacterium-derived carbohydrates, thereby facilitating the phagocytosis of bacteria (4). In addition, mice lacking Mindin were susceptible to influenza virus infection and displayed defective activation of macrophages in response to viral stimuli (6). Recombinant Mindin could interact with viral particles directly and enhanced clearance of viruses (6). These findings show that Mindin can act as a pattern recognition protein for microbial pathogens. In addition to its role in initiating innate immunity, Mindin also functions in T-cell priming and inflammatory cell recruitment after infection. The priming of CD4^+^ T cells by dendritic cells (DCs) was suppressed in Mindin-deficient mice. This might have been caused by the reduced expression of Rho GTPases and inflammatory cytokines in DCs and inefficient engagement of T lymphocytes (7). Moreover, the recruitment of macrophages and neutrophils to the site of inflammation was also inhibited in the mutant mice, demonstrating a role for Mindin in leukocyte migration (5).

The interaction of Mindin with integrin, the critical receptor for ECM proteins, is a determinant for its role in immune regulation (9). Mouse Mindin is a ligand for α4β1 and αMβ2 integrins in the context of recruiting neutrophils (5), while it modulates the expression of inflammatory cytokines and Rho GTPases through α4β1 and α5β1 integrin signaling in DCs (7). In addition, Mac-1 (αMβ2) integrin serves as the opsonic receptor for Mindin. By binding to the αM-I domain of Mac-1 through its spondin domain, Mindin induces the phosphorylation of spleen-associated tyrosine kinase (Syk) and other downstream mitogen-activated protein kinases (MAPKs) to enhance macrophage phagocytosis. This interaction also activates nuclear factor kappa B (NF-κB) p65 nuclear translocation, which ultimately leads to the production of inflammatory cytokines, including tumor necrosis factor (TNF) and interleukin 6 (IL-6) (10). The structural basis of the human Mindin-integrin interaction has been revealed. The wide presence of integrin binding sites in multiple Mindin homologs suggested that the Mindin/integrin axis may be universal among organisms (11).

Despite the importance of Mindin in mammalian innate and adaptive immunity, its role in the immunity of other organisms remains largely unknown. White spot syndrome virus (WSSV) is an enveloped double-stranded DNA virus that has greatly influenced worldwide shrimp farming in the last 30 years (12). When attempting to identify virus-responding genes using transcriptomic analysis in kuruma shrimp (Marsupenaeus japonicus), we observed the significant induction of the Mindin homolog in shrimp. In the present study, Mindin was selected for further functional and mechanistic characterization. We revealed that shrimp Mindin facilitates WSSV infection by activating autophagy, which promotes lipid metabolism to generate energy for viral entry and replication. Therefore, this study reveals the importance of Mindin for host-pathogen interaction and provides a potential target for WSSV prevention in shrimp farming.

## RESULTS

### Mindin responds to and facilitates WSSV infection.

**FIG 1:**
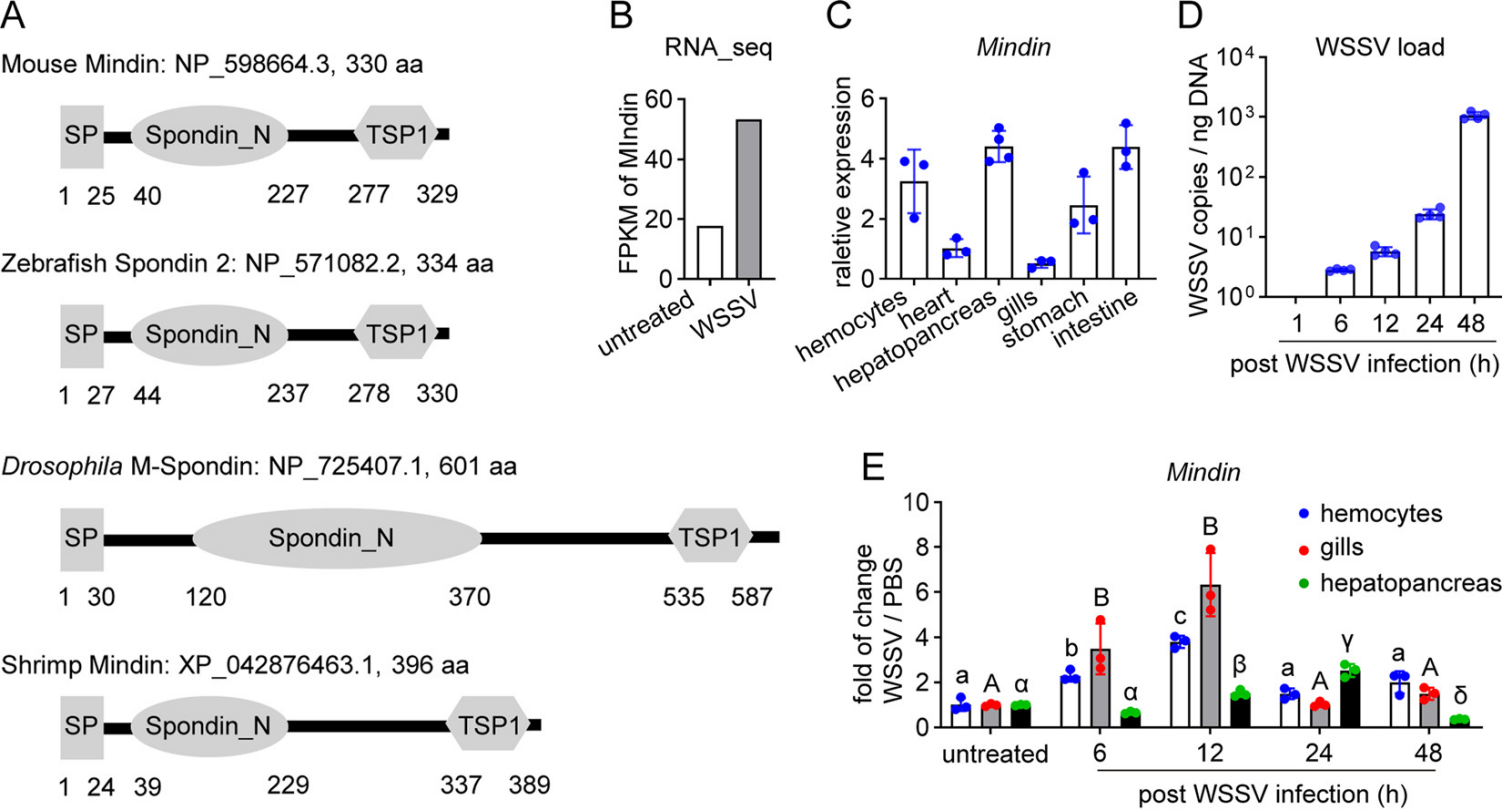
Mindin expression responds to WSSV infection. (A) Architecture arrangement of Mindin family members from mouse, zebrafish, *Drosophila*, and kuruma shrimp. The numbers show the starting and ending residues of a module. aa, amino acids. (B) Variation in abundance of Mindin transcripts after WSSV infection revealed by a transcriptome analysis. Shrimp were infected with a WSSV inoculum (5 × 10^5^ copies). RNA from the gills was extracted 24 h later from 30 animals and subjected to transcriptome sequencing. FPKM value is used to evaluate the abundance of transcripts. (C) Tissue distribution of *Mindin* mRNA. RT-qPCR was performed to detect *Mindin* expression, with *β-actin* as internal reference. Mean ± standard deviations (SD) from three or four independent experiments. (D) Quantification of WSSV load in gills after infection. Shrimp were infected with a WSSV inoculum (5 × 10^5^ copies). Genomic DNA was extracted at certain times after infection and was used to quantify the viral load by qPCR. Mean ± SD from four independent experiments. (E) Expression profiles of *Mindin* mRNA in hemocytes, gills, and hepatopancreas after infection with a WSSV inoculum (5 × 10^5^ copies), analyzed by RT-qPCR. Mean ± SD from three independent experiments. Different letters indicate significant difference (*P* < 0.05) by one-way analysis of variance (ANOVA).

The Mindin family is characterized by the architecture consisting of an N-terminal spondin domain and a C-terminal TSP module. This architecture is conserved from invertebrates to vertebrates (Fig. 1A). By a transcriptome analysis which was performed to screen the WSSV-inducible genes, shrimp Mindin expression was found responding to WSSV infection (Fig. 1B). To confirm the transcriptome sequencing result and determine whether Mindin is involved in the shrimp-WSSV interaction, real-time quantitative reverse transcription-PCR (RT-qPCR) was performed to determine the expression profile of Mindin. As shown in Fig. 1C, Mindin transcripts were distributed in several tested tissues. When shrimp were infected with WSSV (Fig. 1D), Mindin expression was induced (Fig. 1E). Moreover, the induction was detected at the early stage of infection (6 h postinfection [hpi]) in hemocytes and gills (Fig. 1E), the two major target tissues for WSSV infection, indicating that shrimp Mindin might play a role in shrimp-WSSV interaction.

**FIG 2:**
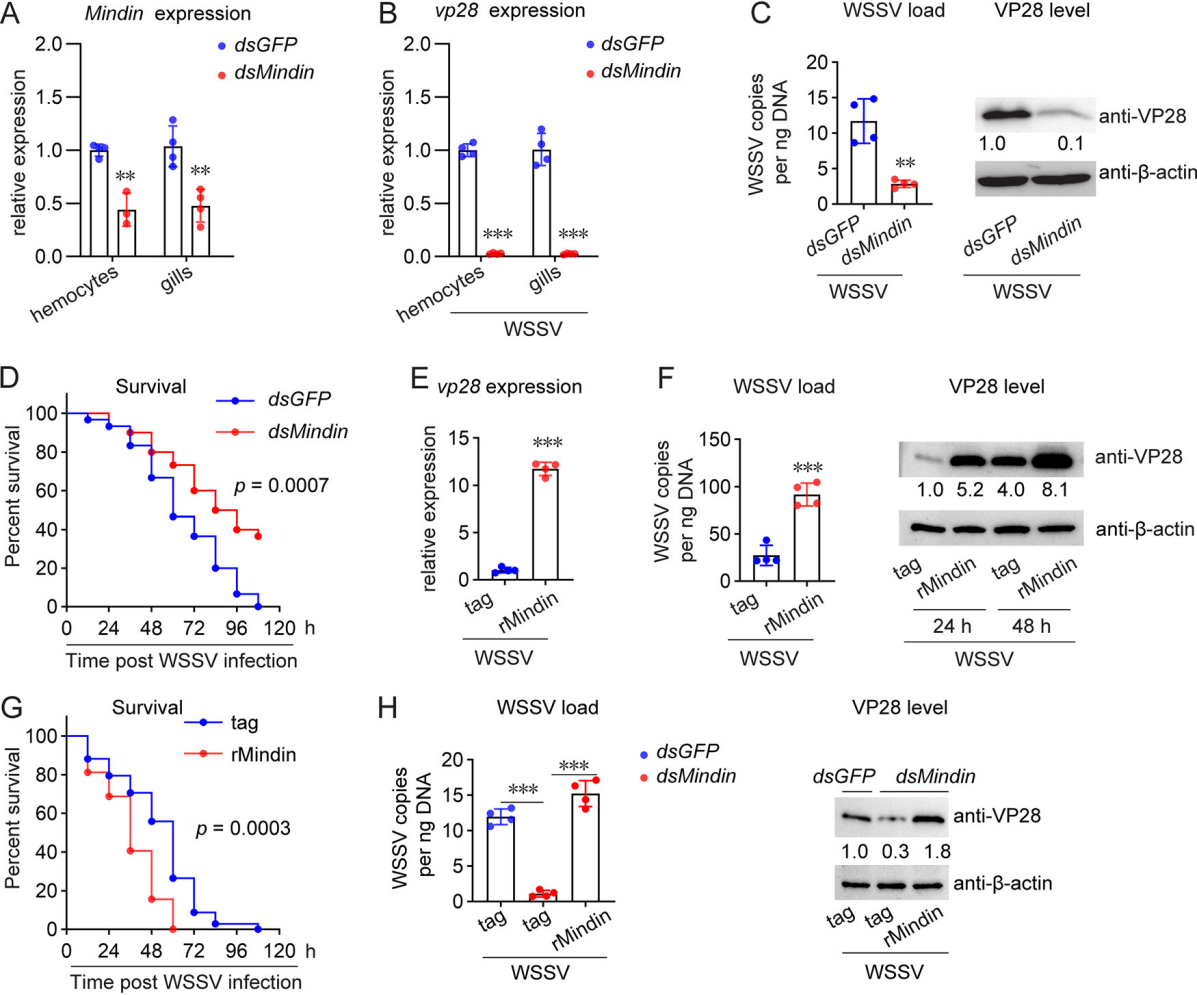
Mindin facilitates WSSV infection. (A) RNAi efficiency of Mindin expression at 48 h after dsRNA injection as assessed using RT-qPCR. (B and C) Effect of *Mindin* knockdown on WSSV infection. WSSV infection (5 × 10^5^ copies) was performed at 48 h after dsRNA injection. *vp28* transcription (B), WSSV load (C, left panel), and VP28 levels (C, right panel) in gills were detected another 24 h later. (D) Effect of *Mindin* knockdown on shrimp survival after WSSV infection. WSSV infection (1 × 10^6^ copies) was performed at 48 h after dsRNA injection. The survival rate was recorded every 12 h for 108 h. *n* = 30. (E and F) Effect of rMindin administration *in vivo* on WSSV infection. Shrimp were injected with 5 μg of rMindin or the control tag and the WSSV inoculum (5 × 10^5^ copies). *vp28* transcription (E), WSSV load (F, left panel), and VP28 levels (F, right panel) were detected 24 h later. (G) Effect of rMindin application on shrimp survival after WSSV infection. Shrimp were injected with 5 μg of rMindin or the control tag and the WSSV inoculum (1 × 10^6^ copies). The survival rate was recorded every 12 h for 108 h. *n* = 30. (H) Restoring the *Mindin* knockdown suppressed WSSV infection by rMindin. Shrimp were injected with 5 μg of rMindin or the control tag and the WSSV inoculum (5 × 10^5^ copies) at 48 h after dsRNA injection. WSSV load (left panel) and VP28 levels (right panel) were detected another 24 h later. All bar chart data are shown as the mean ± SD from three or four replicates. ***, *P* < 0.001, and **, 0.001 < *P* < 0.01, as determined using Student’s *t* test. The survival data are representative of three biological replicates and analyzed by log rank (Mantel-Cox) test. The Western blotting images are representative of three independent replicates, and the numbers indicate the relative band intensities (VP28/β-actin).

To reveal the role of Mindin in WSSV infection, RNA interference (RNAi) was performed to knock down Mindin expression. As shown in Fig. 2A, the expression levels of *Mindin* in hemocytes and gills were significantly downregulated by double-stranded RNA (dsRNA) application (Fig. 2A). WSSV inoculation was then performed in dsRNA-treated shrimp to determine whether *Mindin* knockdown would influence viral infection. The results showed that decreased expression of *Mindin* led to reduced transcription of *vp28*, which encodes the most abundant structural protein of WSSV (Fig. 2B). Significant reductions in the viral load and VP28 levels were also observed (Fig. 2C and see Fig. S1A in the supplemental material). Moreover, the mortality caused by WSSV infection was suppressed by *Mindin* knockdown (Fig. 2D), suggesting that Mindin might facilitate WSSV infection. To verify the results, recombinant Mindin (rMindin) was produced and administered to shrimp hemocoels to generate an overexpression-like effect. rMindin application, which did not cause a toxic effect on shrimp survival (Fig. S1B), significantly increased WSSV *vp28* transcription (Fig. 2E), the viral load, and VP28 levels (Fig. 2F and Fig. S1C). Furthermore, rMindin administration accelerated shrimp death caused by WSSV infection, and the survival rate after WSSV infection in the rMindin-administered group was always lower than that of the control group (Fig. 2G). These results confirmed the role of Mindin in facilitating WSSV infection. We also administered rMindin after *Mindin* knockdown and found that rMindin administration restored the inhibitory effect by stopping the decrease of viral load and VP28 level caused by *Mindin* knockdown (Fig. 2H). Collectively, the above data demonstrated that Mindin facilitates WSSV infection.

10.1128/mbio.02919-22.1 FIG S1 Mindin facilitates WSSV infection. (A) Dose-dependent effect of Mindin dsRNA on WSSV infection. Serial doses of Mindin dsRNA were injected into shrimp hemocoel. WSSV infection (5 × 10^5^ copies) was performed 48 h later. WSSV load (left panel) and VP28 levels (right panel) in gills were detected another 24 h later. (B) Influence of rMindin application on shrimp survival. Shrimp were injected with 5 μg of rMindin or the control tag. The survival rate was recorded. *n* = 30. ns, no significant difference, log rank (Mantel-Cox) test. (C) Dose-dependent effect of rMindin on WSSV infection. Shrimp were injected with serial amounts of rMindin or control tag, and the WSSV inocula (5 × 10^5^ copies). WSSV load (left panel) and VP28 levels (right panel) in gills were detected another 24 h later. The bar chart data are shown as the mean ± SD from four replicates. ***, *P* < 0.001, and ns, no significant difference, by Student’s *t* test. The survival data are representative of two biological replicates and analyzed by log rank (Mantel-Cox) test. The Western blotting images are representative of three independent replicates, and the numbers indicate the relative band intensities (VP28/β-actin). Download FIG S1, TIF file, 2.4 MB. Copyright © 2023 Gao et al. 2023 Gao et al. https://creativecommons.org/licenses/by/4.0/ This content is distributed under the terms of the Creative Commons Attribution 4.0 International license.

### Mindin benefits virus infection by facilitating WSSV entry.

**FIG 3:**
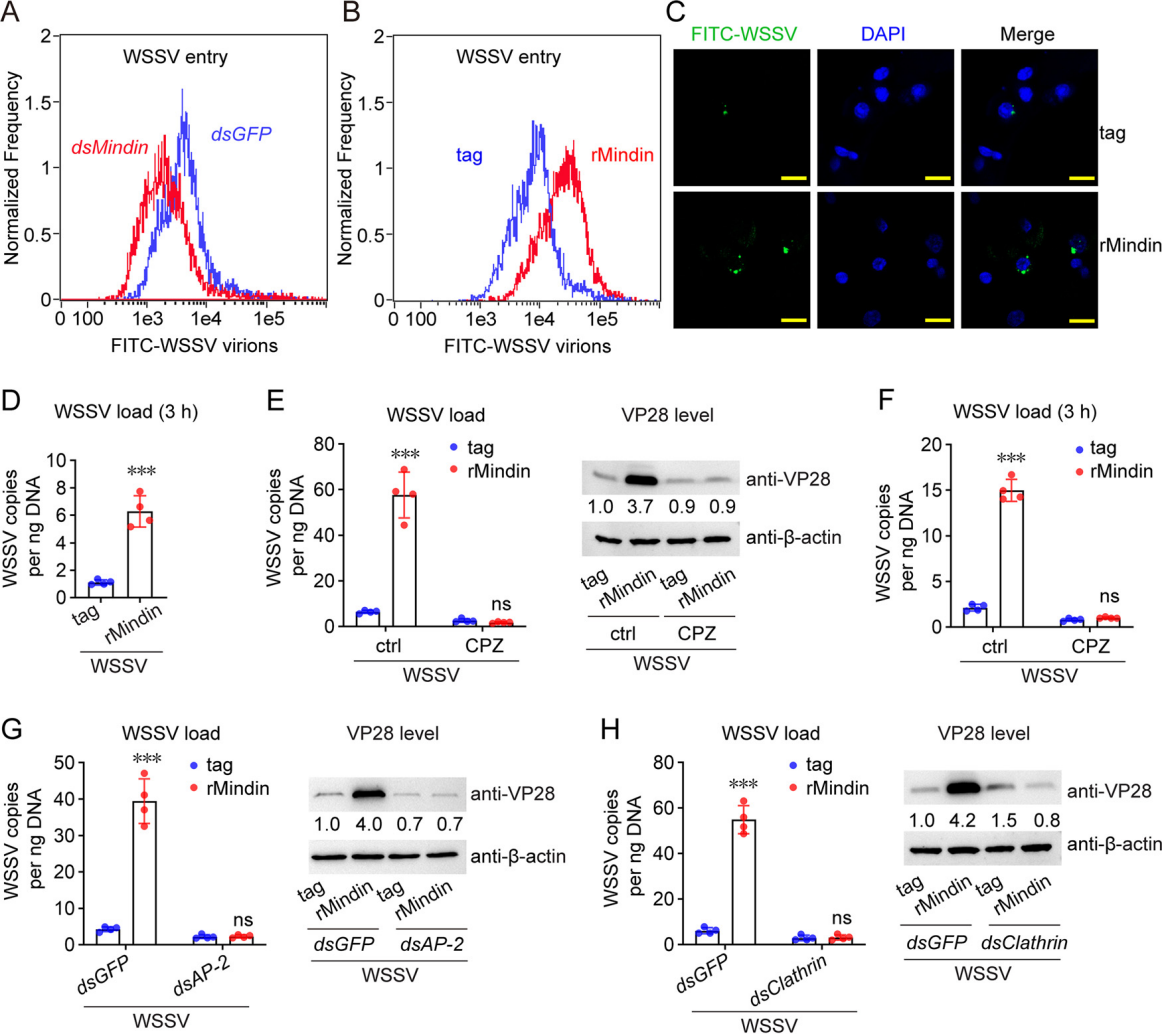
Mindin facilitates WSSV entry and replication. (A) Mindin facilitates WSSV entry as revealed by RNAi assay. FITC-labeled virions (10^6^ copies) were injected into shrimp hemocoels at 48 h after dsRNA injection. Shrimp hemocytes were collected and analyzed using flow cytometry. (B to D) Mindin facilitates WSSV entry as revealed by rMindin application. FITC-labeled virions (10^6^ copies) were injected into shrimp hemocoels together with rMindin or the control tag (5 μg). Shrimp hemocytes were collected and analyzed using flow cytometry (B) and confocal microscopy (C) 1 h later. DAPI was used to stain the nuclei. Bar, 10 μm. Genomic DNA was also extracted from hemocytes at 3 h after injection to monitor the number of virions that had entered the hemocytes (D). (E) Influence of clathrin-mediated endocytosis inhibition by using chlorpromazine (CPZ) on Mindin-enhanced WSSV infection. CPZ was applied *in vivo* at a dose of 50 μg/shrimp to inhibit clathrin-mediated endocytosis. Injection of rMindin (5 μg) and the WSSV inoculum (5 × 10^5^ copies) was performed 2 h later. The WSSV load (left panel) and VP28 expression (right panel) in gills were detected another 24 h later. (F) Influence of CPZ application on Mindin-enhanced WSSV entry. Injection of rMindin and the WSSV inoculum was performed at 2 h after CPZ application. Genomic DNA was extracted from hemocytes at 3 h after injection to monitor the number of virions that had entered the hemocytes. (G and H) Influence of *Clathrin* knockdown (G) or *AP-2* knockdown (H) on Mindin-enhanced WSSV infection. Injection of rMindin (5 μg) and the WSSV inoculum (5 × 10^5^ copies) was performed 48 h after dsRNA injection. The WSSV load (left panel) and VP28 expression (right panel) in gills were detected 24 h later. All bar chart data are shown as the mean ± SD from three or four replicates. ***, *P* < 0.001, and ns, no significant difference, Student’s *t* test. All flow cytometry images, microscopy images, and Western blotting images are representative of three independent replicates. The numbers in blotting figures indicate the relative band intensities (VP28/β-actin).

Next, how Mindin benefits WSSV infection was investigated. Fluorescein isothiocyanate (FITC)-labeled WSSV virions were injected into shrimp hemocoels after *Mindin* knockdown. Flow cytometry was performed to detect the FITC signal in hemocytes, which represented viral entry, at the initial phase of infection (1 hpi). As shown in Fig. 3A, *Mindin* knockdown suppressed WSSV entry. To confirm this result, the labeled virions together with rMindin were delivered into shrimp hemocoels. The flow cytometry result showed that rMindin application enhanced WSSV entry (Fig. 3B). Meanwhile, confocal microscopy showed that the amount of FITC-labeled virions in hemocytes was larger in the rMindin group than in the control group (Fig. 3C and Fig. S2). We also quantified the viral load in the hemocytes before the new virus was produced, and the data confirmed that rMindin enhanced WSSV entry (Fig. 3D).

10.1128/mbio.02919-22.2 FIG S2 Statistical analysis of the viral entry in hemocytes. ImageJ software was used to determine the number of virus-positive hemocytes, and the percentage of virus-positive hemocytes was calculated. The data are shown as the mean ± SD from four replicates. ***, *P* < 0.001, Student’s *t* test. Download FIG S2, TIF file, 0.2 MB. Copyright © 2023 Gao et al. 2023 Gao et al. https://creativecommons.org/licenses/by/4.0/ This content is distributed under the terms of the Creative Commons Attribution 4.0 International license.

To further confirm that Mindin promotes WSSV entry, we treated shrimp with chlorpromazine (CPZ), an inhibitor of clathrin-mediated endocytosis (CME), which is the major means of WSSV entry (13), to check whether the function of Mindin was restricted. CPZ application did not cause an obvious toxic effect on shrimp survival (Fig. S3A). The results showed that Mindin-promoted WSSV infection disappeared after CPZ treatment (Fig. 3E). Moreover, the inhibitory effect of CPZ was dose dependent (Fig. S3B). We also detected the viral load at 3 hpi before the new virus was produced. As shown in Fig. 3F, CPZ application indeed inhibited Mindin-mediated viral entry. We also silenced the expression of *Clathrin* and *AP-2* (encoding activator protein 2), two key components for CME, to determine whether the effect of Mindin was influenced. We found that knockdown of *Clathrin* or *AP-2* inhibited the Mindin-enhanced WSSV infection (Fig. 3G and H). Therefore, these results demonstrated that Mindin promotes WSSV invasion by facilitating its entry.

10.1128/mbio.02919-22.3 FIG S3 Dose-dependent inhibitory effect of CPZ on Mindin-enhanced WSSV infection. (A) Influence of CPZ application on shrimp survival. Shrimp were injected with CPZ (50 μg) or the dissolvent as the control. The survival rate was recorded. *n* = 30. The survival data are representative of two biological replicates. ns, no significant difference, log rank (Mantel-Cox) test. (B) Dose-dependent effect of CPZ application on Mindin-enhanced WSSV infection. Shrimp were treated with serial amounts of CPZ. Injection of rMindin (5 μg) and the WSSV inoculum (5 × 10^5^ copies) was performed 2 h later. The WSSV load (left panel) and VP28 expression (right panel) in gills were detected another 24 h later. The bar chart data are shown as the mean ± SD from four replicates. ***, *P* < 0.001, and *, 0.01 < *P* < 0.05, by Student’s *t* test. The Western blotting images are representative of three independent replicates, and the numbers indicate the relative band intensities (VP28/β-actin). Download FIG S3, TIF file, 1.5 MB. Copyright © 2023 Gao et al. 2023 Gao et al. https://creativecommons.org/licenses/by/4.0/ This content is distributed under the terms of the Creative Commons Attribution 4.0 International license.

### Mindin regulates lipid metabolism to facilitate WSSV infection.

**FIG 4:**
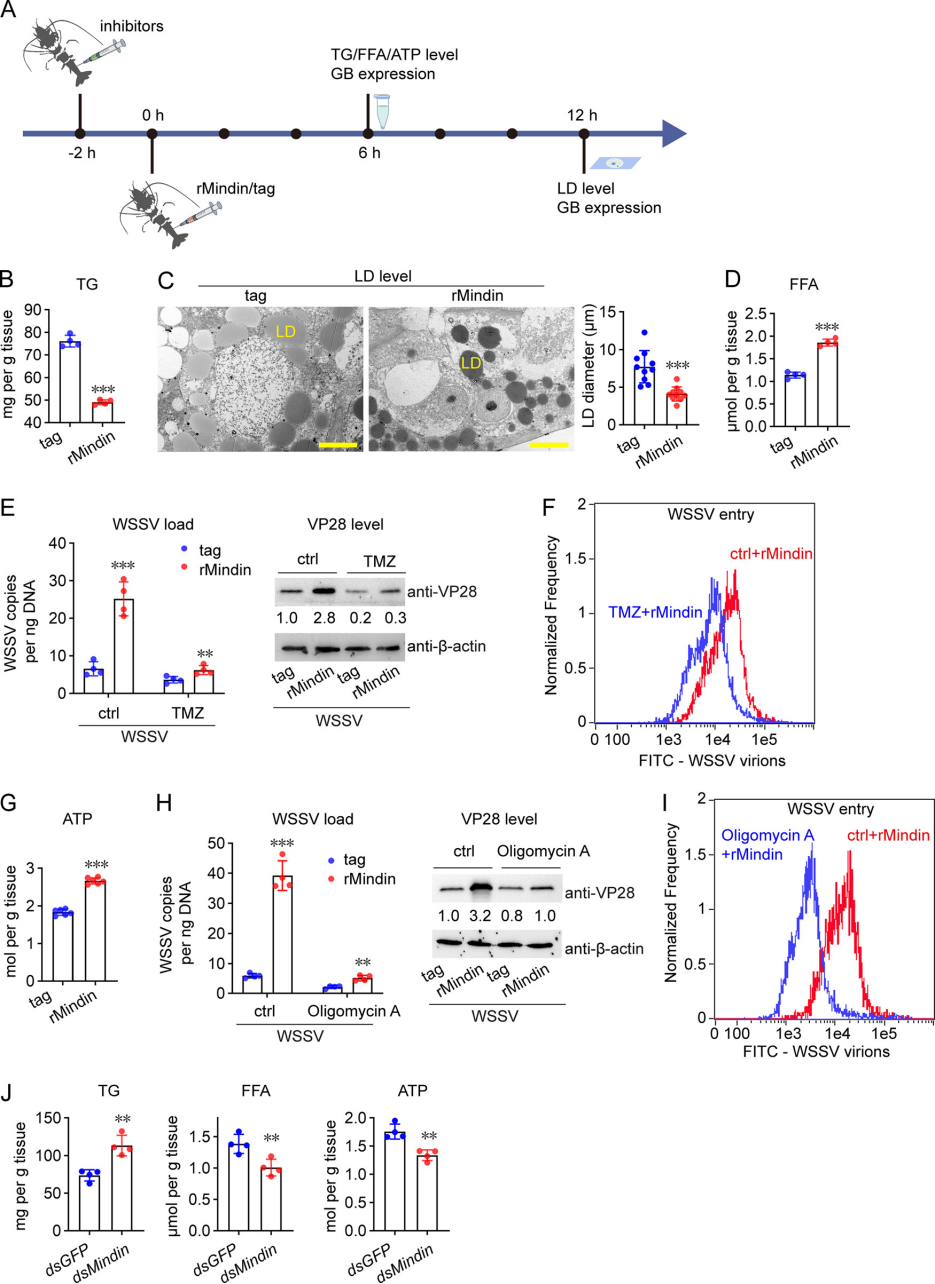
Mindin regulates lipid metabolism to facilitate WSSV infection. (A) Schematic illustration of the experimental procedures. (B) Effect of Mindin on the TG content. The hepatopancreas was collected 6 h after rMindin or tag (5 μg) administration to determine the TG level by using a commercial TG content detection kit. (C) Effect of Mindin on LD volume. The hepatopancreas was collected 12 h after rMindin or tag (5 μg) administration, processed, and observed under a transmission electron microscope. Bar, 10 μm. Right panel, quantification of LD diameter by ImageJ software. (D) Effect of Mindin on the FFA content. The FFA level in hepatopancreas was detected at 6 h after rMindin administration by using an FFA content detection kit. (E) Influence of FFA hydrolysis inhibition by using trimetazidine (TMZ) on Mindin-enhanced WSSV replication. TMZ was applied *in vivo* at a dose of 5 μg/shrimp to inhibit fatty acid oxidation. Injection of rMindin and the WSSV inoculum was performed at 2 h after TMZ application. The WSSV load (left panel) and VP28 expression (right panel) in gills were detected 24 h later. (F) Influence of FFA hydrolysis inhibition on Mindin-enhanced WSSV entry. FITC-labeled virions (10^6^ copies) were injected into shrimp hemocoels together with rMindin or the control tag at 2 h after TMZ application. WSSV entry was detected by flow cytometry assay 1 h later. (G) Effect of Mindin on the ATP content. ATP levels in hepatopancreas were detected at 6 h after rMindin application by using an enhanced ATP assay kit. (H) Influence of ATP synthesis inhibition by using oligomycin A on Mindin-enhanced WSSV replication. Oligomycin A was applied *in vivo* at a dose of 1 μg/shrimp to inhibit ATP synthesis. Injection of rMindin and the WSSV inoculum was performed at 2 h after oligomycin A application. The WSSV load (left panel) and VP28 expression (right panel) in gills were detected 24 h later. (I) Influence of ATP synthesis inhibition on Mindin-enhanced WSSV entry. FITC-labeled virions (10^6^ copies) were injected into shrimp hemocoels together with rMindin or the control tag at 2 h after oligomycin A application. WSSV entry was detected using flow cytometry 1 h later. (J) Effect of Mindin knockdown on the level of TG, FFA, and ATP. The hepatopancreas was collected at 48 h after Mindin dsRNA application to determine the TG, FFA, and ATP levels by using the corresponding kits. All bar chart data are shown as mean ± SD from three or four replicates. ***, *P* < 0.001, and **, 0.001 < *P* < 0.01, Student’s *t* test. All flow cytometry images, microscopy images, and Western blotting images are representative of three independent replicates. The numbers in blotting panels indicate the relative band intensities (VP28/β-actin).

It was reported previously that Mindin regulates the lipid metabolism (14). Therefore, we investigated whether this is responsible for Mindin’s effect on WSSV infection in shrimp. Triglycerides (TGs) are the main form of fat storage. First, we quantified TG levels using a colorimetric assay after administering rMindin into shrimp hemocoels (Fig. 4A). As shown in Fig. 4B, rMindin application caused significant decrease in the TG level compared with that in the controls. TGs are the main component of lipid droplets (LDs); therefore, we also monitored the size of LDs in the rMindin-treated shrimp and observed by transmission electron microscopy that rMindin application led to a reduction in the LD volume compared with that in the controls (Fig. 4C). These results suggested that Mindin might mediate lipid utilization in shrimp. Then, we measured the content of free fatty acids (FFAs), which are the hydrolysis products of TGs. The results showed that rMindin significantly increased the FFA content compared with that in the controls (Fig. 4D). To determine whether FFAs play a role in Mindin-enhanced WSSV infection, trimetazidine (TMZ) was applied in shrimp to inhibit fatty acid oxidation, before inoculation with WSSV and rMindin. As shown in Fig. 4E, TMZ treatment significantly reduced the ability of Mindin to promote WSSV replication, as revealed by the viral load and the VP28 level, compared with those in the controls. Viral entry was also detected, and the results showed that Mindin-mediated WSSV entry was reduced when fatty acid oxidation was blocked compared with that in the controls (Fig. 4F).

FFAs undergo β-oxidation for ATP production, and the virus infection cycle is an energy-consuming process; therefore, we hypothesized that Mindin promotes ATP production to facilitate virus infection. To test this hypothesis, we measured the variation in ATP levels after rMindin application. The data showed that the ATP level in the rMindin group was higher than that in the control group (Fig. 4G). To determine whether ATP generation was really related to Mindin-enhanced WSSV infection, oligomycin A, an inhibitor of ATP synthesis, was preinjected into the shrimp before inoculation with WSSV and rMindin. The results showed that both Mindin-enhanced WSSV replication (Fig. 4H) and entry (Fig. 4I) were suppressed upon inhibition of ATP production. To confirm the data obtained by using the overexpression test, we also detected the effect of *Mindin* knockdown on the lipid metabolism. As shown in Fig. 4J, the TG level was upregulated, while the FFA level and ATP level were downregulated, by *Mindin* knockdown. Together, these results suggested that Mindin contributes to WSSV infection by regulating lipid utilization, thereby ensuring the energy supply for viral entry and replication.

### Mindin regulates lipid metabolism by activating autophagy.

**FIG 5:**
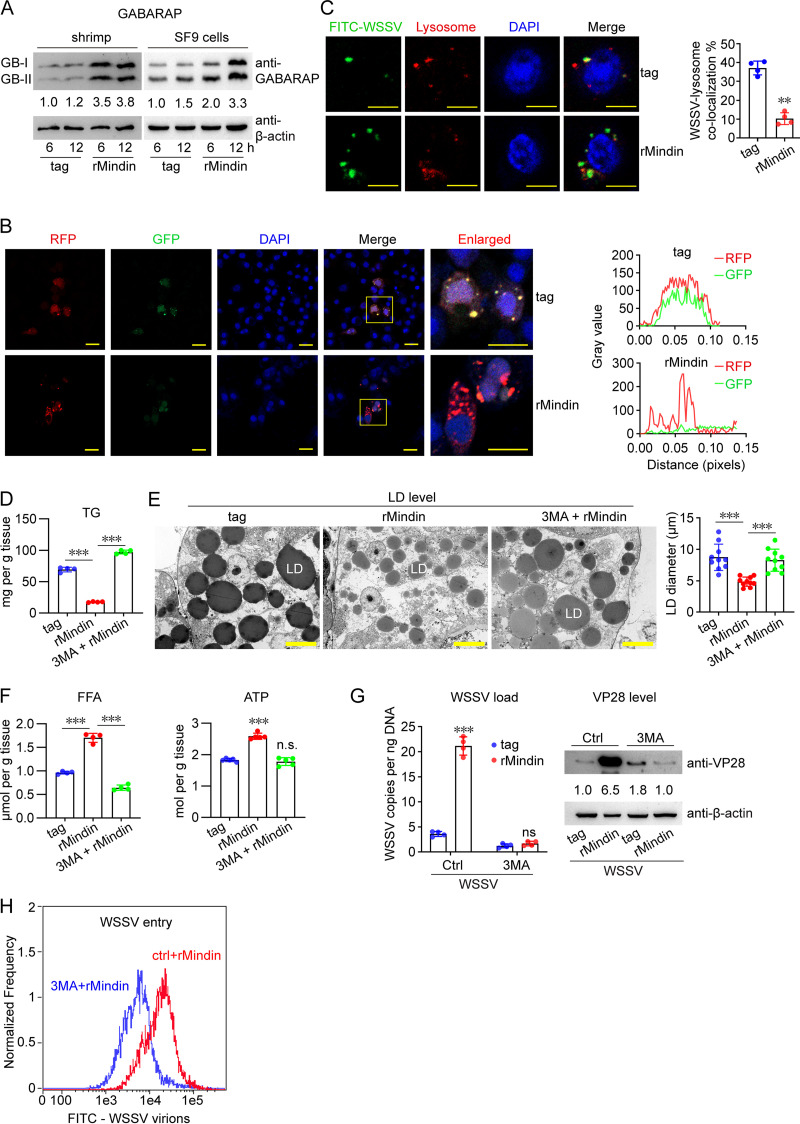
Mindin activates autophagy for lipid metabolism and WSSV infection. (A) Induction of GABARAP-II by rMindin. rMindin (5 μg) was injected into shrimp or added to the medium of Sf9 cells. GABARAP-II level in shrimp hemocytes or Sf9 cells was detected 6 h and 12 h later. (B) Induction of autophagosome formation in Sf9 cells by rMindin. The pIEx-4-RFP-GFP-LC3-His reporter plasmid was transformed into Sf9 cells. rMindin or the control tag (5 μg) was added into the medium 48 h later to induce autophagy. Cells were collected another 12 h later, and the nucleus was stained with DAPI. Fluorescence was visualized under a confocal microscope. The merged red color, but not merged yellow color, indicates autophagosome formation. Bar, 20 μm. (Right panel) The fluorescence intensity was quantified across a line spanning the whole cell by using ImageJ software. (C) Inhibition of the colocalization of WSSV virions and lysosomes by rMindin. Hemocytes were collected at 3 h after the injection of rMindin and the FITC-labeled virions (10^6^ copies) and observed under a confocal microscope. The nucleus was stained by DAPI, and lysosomes were tracked by LysoBrite red reagent. Bar, 20 μm. (Right panel) Colocalization of WSSV and lysosome quantified using ImageJ software. **, 0.001 < *P* < 0.01, Student’s *t* test. (D to F) Influence of autophagy inhibition by using 3-methyladenine (3-MA) on Mindin-induced variation of TGs (D), LDs (E), and FFAs and the ATP level (F). 3-MA was administered with the dose of 5 μg/animal. rMindin (5 μg) application was performed at 2 h after 3-MA administration. TGs, FFAs, and the ATP levels in hepatopancreas were detected 6 h later, while LDs were detected 12 h later. Bar, 10 μm. Quantification of LD diameter was performed by ImageJ software. (G) Influence of autophagy inhibition on Mindin-enhanced WSSV replication. Injection of rMindin (5 μg) and the WSSV inoculum (5 × 10^5^ copies) was performed at 2 h after 3-MA application. The WSSV load (left panel) and VP28 level (right panel) in gills were detected 24 h later. (H) Influence of autophagy inhibition on Mindin-enhanced WSSV entry. FITC-labeled virions (10^6^ copies) were injected into shrimp hemocoels together with rMindin or the control tag (5 μg) at 2 h after 3-MA application. WSSV entry was detected using flow cytometry 1 h later. All bar chart data are shown as the mean ± SD from three or four replicates. ***, *P* < 0.001, and ns, no significant difference, Student’s *t* test. All Western blotting images, microscopy images, and flow cytometry images are representative of three independent replicates. The numbers in blotting panels indicate the relative band intensities (VP28/β-actin).

Next, we wanted to know how Mindin promotes lipid utilization for virus infection. Autophagy plays a pivotal role in lipid metabolism and the degradation of LDs (15); therefore, we investigated whether Mindin regulates lipid utilization by activating autophagy. First, the involvement of Mindin in autophagy was clarified. Induction of γ-aminobutyric acid receptor-associated protein II (GABARAP-II) is a marker of autophagy activation (16). Its level after rMindin application was detected. Results showed that rMindin application induced the generation of GABARAP-II in shrimp (Fig. 5A). Moreover, when rMindin was administered into the culture medium of Sf9 cells, an insect cell line able to support WSSV infection and replication (17), the induction of GABARAP-II was also initiated (Fig. 5A). This suggested that Mindin might induce autophagy in both shrimp and Sf9 cells. To confirm this finding, microtubule-associated protein 1 light chain 3 (LC3) fused with red and green fluorescence proteins (RFP-GFP-LC3) was overexpressed in Sf9 cells to monitor Mindin-activated autophagy. Autophagy activation causes localization of LC3 to lysosomes, and GFP can be broken down by the acidic hydrolase present in the lysosomes; therefore, only red, but not merged yellow, fluorescence would be observed after the formation of autophagosomes (18). As shown in Fig. 5B, merged yellow signals were observed in the control group, while only the RFP signal was observed in the rMindin group, which suggested that the formation of autophagic lysosomes occurred after rMindin treatment. These results demonstrated that Mindin might activate autophagy. Multiple studies have shown that autophagy activation induces lysosomal degradation of viruses (19, 20); therefore, we next clarified whether Mindin-activated autophagy would lead to WSSV degradation. As shown in Fig. 5C, compared with that in the control, rMindin caused less colocalization of virus and lysosomes, indicating that Mindin could protect WSSV from lysosomal degradation. Although the mechanism by which WSSV escapes from autophagy-mediated degradation is unknown, this result again suggested the beneficial role of Mindin in WSSV infection.

Next, the requirement of autophagy for TG depletion was tested. 3-Methyladenine (3-MA), an inhibitor of autophagy, was used to decrease autophagic activity to check whether Mindin-mediated lipid utilization was impaired. As shown in Fig. 5D and E, inhibiting autophagy prevented the depletion of TGs and LDs caused by rMindin application. Moreover, inhibition of autophagy limited the increase of FFA and ATP levels induced by rMindin (Fig. 5F).

Having revealed that Mindin-activated autophagy is essential for lipid utilization and energy production, we next determined whether this autophagy contributed to Mindin-enhanced WSSV infection. As shown in Fig. 5G, the ability of Mindin to facilitate WSSV replication was inhibited by 3-MA, as revealed by the viral load and VP28 level. More importantly, Mindin-mediated WSSV entry was also impaired by autophagy inhibition (Fig. 5H). Therefore, these results collectively supported the view that Mindin facilitates WSSV infection by activating autophagy and subsequent lipid utilization.

### Integrin is essential for the role of Mindin in WSSV infection.

**FIG 6:**
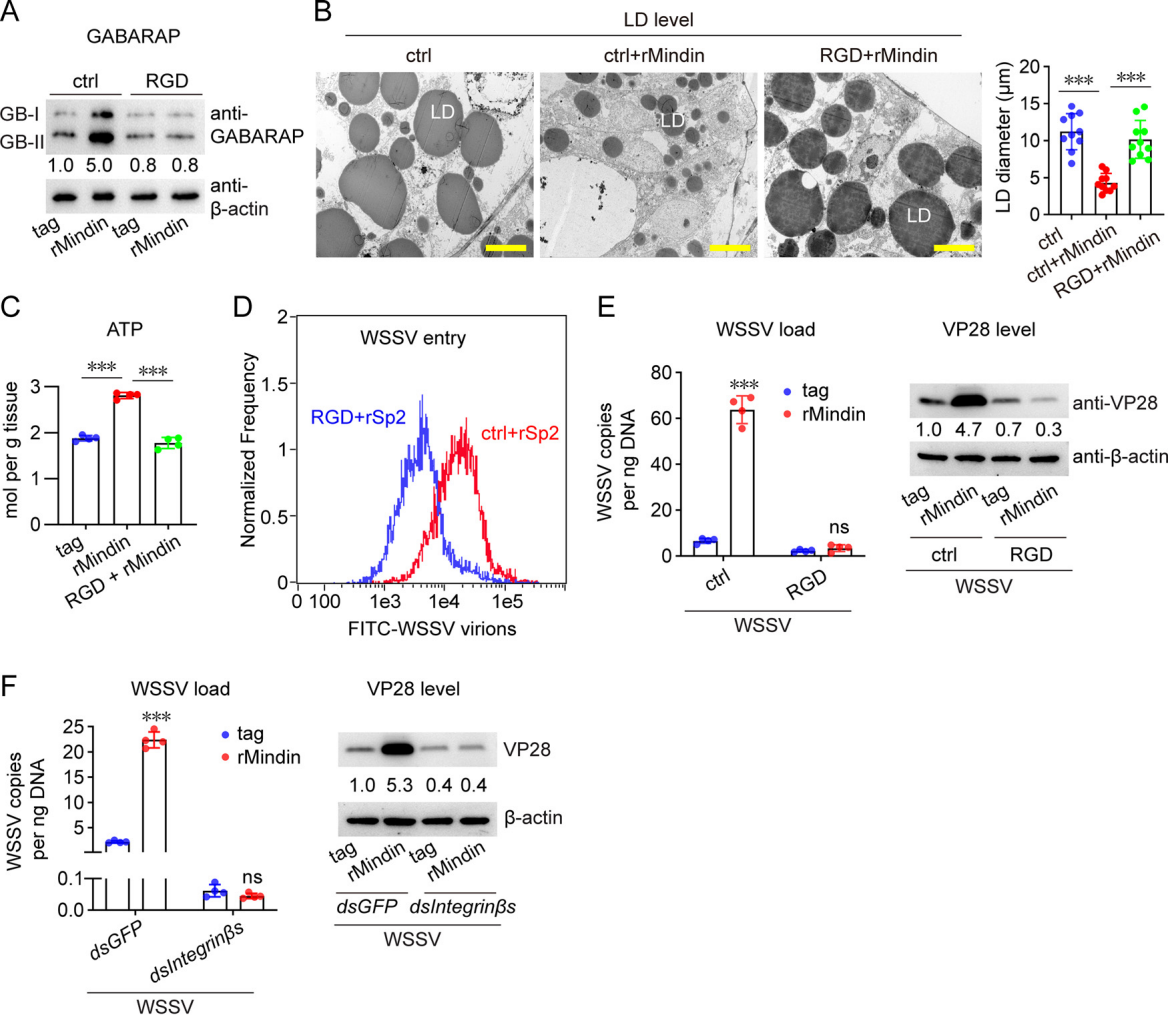
Integrin is essential for Mindin-enhanced WSSV infection. (A) Influence of integrin signaling inhibition by using RGD peptide on Mindin-induced autophagy. RGD peptide was administered at the dose of 5 μg/shrimp to block integrin signaling. rMindin injection (5 μg) was performed at 2 h after RGD peptide application. GABARAP expression in hemocytes was detected another 6 h later. (B and C) Influence of integrin signaling inhibition on Mindin-induced variation of LDs and the ATP level. rMindin injection was performed at 2 h after RGD peptide application. The hepatopancreas was collected to observe the LDs another 12 h later (B) or to detect the ATP level another 6 h later (C). Bar, 10 μm. Quantification of LD diameter was performed by ImageJ software. (D) Influence of integrin signaling inhibition on Mindin-enhanced WSSV entry. FITC-labeled virions (10^6^ copies) were injected into shrimp hemocoels together with rMindin or the control tag (5 μg) at 2 h after RGD peptide application. WSSV entry was detected using flow cytometry 1 h later. (E) Influence of integrin signaling inhibition on Mindin-enhanced WSSV replication. Injection of rMindin and WSSV inoculum (5 × 10^5^ copies) was performed at 2 h after RGD peptide application. WSSV load (left panel) and VP28 expression (right panel) in gills were detected 24 h later. (F) Effect of integrin knockdown on Mindin-enhanced WSSV infection. Injection of rMindin and WSSV inoculum (5 × 10^5^ copies) was performed at 48 h after dsRNA injection. WSSV load (left panel) and VP28 expression (right panel) in gills were detected 24 h later. All bar chart data are shown as the mean ± SD from three or four replicates. ***, *P* < 0.001, and ns, no significant difference, Student’s *t* test. All Western blotting images, microscopy images, and flow cytometry images are representative of three independent replicates. The numbers in blotting panels indicate the relative band intensities (VP28/β-actin).

As an ECM protein, Mindin needs to interact with membrane receptors to exert its function (21). Studies have shown that Mindin acts as a ligand for integrins (5, 11). Therefore, we detected whether integrin is required for Mindin’s role in WSSV infection. We first detected the interaction between Mindin and integrin in shrimp. As shown in Fig. S4, physical interaction between Mindin and integrin β subunits was revealed by yeast two-hybrid assay. Moreover, rMindin could interact with the surface of hemocytes, and the interaction was blocked after integrin signaling inhibition or integrin expression knockdown (Fig. S4). These data suggested that integrin might serve as the functional receptor for Mindin in shrimp. Afterward, the RGD peptide was administered to shrimp to suppress integrin signaling. As shown in Fig. 6A, RGD application suppressed the induction of GABARAP-II caused by rMindin, suggesting that the Mindin-activated autophagy was dependent on integrin. After RGD application, the reduction in LD volume caused by rMindin was restored (Fig. 6B), while the induction of ATP by rMindin was impaired (Fig. 6C), indicating the necessity of integrin for Mindin-mediated lipid utilization and energy generation. Moreover, we observed that Mindin-mediated WSSV entry was suppressed upon RGD administration (Fig. 6D). The enhancement of WSSV replication level by Mindin was also impaired, as revealed by the viral load and VP28 levels (Fig. 6E), which also indicated the requirement of integrin signaling for the function of Mindin in WSSV infection.

10.1128/mbio.02919-22.4 FIG S4 Mindin interacts with integrin. (A) Physical interaction between Mindin and the extracellular portion of integrin β subunits. The fragments encoding Mindin and the extracellular portion of integrin β subunits were ligated into indicated vectors. The recombinant vectors were transformed into yeast. The transformants were grown on DDO (Leu^−^ Trp^−^) and QDO/X (Ade^−^ Leu^−^ Trp^−^ His^−^ 5-bromo-4-chloro-3-indolyl-β-d-galactopyranoside [X-Gal]^+^) medium. (B) Interaction of rMindin with shrimp hemocytes through integrin. Shrimp hemocytes were collected at 2 h after RGD peptide administration or at 48 h after dsRNA application and incubated with rMindin at 25°C for 1 h. Bound proteins were detected by using the anti-6His antibodies and the fluorescent secondary antibodies. The hemocytes were observed under the Zeiss LSM 900 confocal microscope. Bar, 10 μm. The images are representative of three independent replicates. Download FIG S4, TIF file, 2.8 MB. Copyright © 2023 Gao et al. 2023 Gao et al. https://creativecommons.org/licenses/by/4.0/ This content is distributed under the terms of the Creative Commons Attribution 4.0 International license.

To verify the data obtained using the RGD peptide, we used a mixture of dsRNAs specific for the three integrin β subunits in shrimp to suppress their expression. The data showed that the integrin β knockdown resulted in the loss of Mindin-promoted virus replication and VP28 expression (Fig. 6F). Together, these results suggested that Mindin depends on integrin to activate autophagy and lipid utilization, thus facilitating WSSV infection.

## DISCUSSION

Mindin is highly expressed in the ECM and was initially regarded as important for tissue development. For example, secreted *Drosophila* M-spondin specifically accumulates in the site of muscle attachment, where it may mediate the anchoring of muscle cells on the epidermis (3). Two zebrafish Mindins are concentrated in tightly condensed ECM structures and may modulate cell-matrix interactions, including morphogenetic processes (22). Rat Mindin can promote the adhesion and neurite outgrowth of embryonic hippocampal and sensory neurons, as revealed by an *in vitro* assay using the recombinant protein (2). However, mutations of M-spondin did not generate any obvious defects in *Drosophila* muscle attachment (3). Moreover, ectopic expression of zebrafish Mindins in early embryos had no apparent effect on embryonic development (22). In addition, there is no report of any gross developmental abnormalities in Mindin-deficient or Mindin-transgenic mice (8, 10). Therefore, the role of Mindin should be reconsidered. Aside from the initial assumption, increasing studies have clearly demonstrated that Mindin is important for the host immune response. It has been suggested that Mindin participates in host-pathogen interaction is several ways, namely, by recognizing microbial pathogens for phagocytosis, by mediating certain signaling transduction and cytokine production, and by adhering and recruiting inflammatory cells (8). The studies mentioned above were all performed using mice as the model. The role of Mindin in nonmammal animals is unknown. Here, we revealed that shrimp Mindin activates autophagy and lipid utilization, thereby facilitating WSSV infection. This study uncovered a new role in immunity for the Mindin family by using shrimp-WSSV interaction as a model and provides new insights into the significance of this family in immunity.

Autophagy is an evolutionarily conserved lysosomal degradation process (23). It is a double-edged sword during viral infection (24). Autophagy can restrict virus infection. For example, autophagy mediates host detection of vesicular stomatitis virus and Sendai virus by transporting cytosolic viral replication intermediates into lysosomes for Toll-like receptor 7 recognition, and this mechanism also leads to alpha interferon secretion in plasmacytoid dendritic cells (25); the autophagy receptor p62 is able to directly bind a capsid protein of Sindbis virus and target it to the autophagosome for clearance (20). To deal with these host responses, viruses have evolved strategies to evade autophagic degradation. For instance, HIV-1 Nef can inhibit autophagosome maturation by interacting with Beclin 1 to sequester transcription factor EB, a key factor for autophagy and lysosome biogenesis (26, 27). In addition, viruses can also manipulate the autophagy pathway to support their entry, replication, and release. The picornavirus foot-and-mouth disease virus induces autophagosomes to facilitate its entry (28). Poliovirus infection leads to the formation of autophagosomes, which might provide a membranous scaffold for viral RNA replication complexes (29). Picornaviruses, as nonenveloped RNA viruses, also exploit phosphatidylserine-enriched autophagosomes for package and release (30). A study of WSSV showed that autophagy can restrict viral fusion and uncoating to inhibit virus infection in red claw crayfish (31). However, other studies showed that WSSV entry correlates positively with autophagic activity, and inhibition of autophagy causes decreased viral replication in both crayfish and kuruma shrimp (13, 32). Therefore, although autophagy is an antiviral response against WSSV to a certain degree, WSSV has also evolved strategies to evade or even exploit the autophagy pathway to its own advantage. Thus, the finding in the current study that autophagy activates lipid utilization and energy supply to facilitate WSSV entry and replication established a new mechanism for autophagy in WSSV infection.

LDs are the primary lipid reservoir in eukaryotic cells (33). In a starving cell, induction of autophagy regulates intracellular lipid stores by degrading LDs and delivering fatty acids to mitochondria for β-oxidation, a critical process for ATP production (15). The virus infection cycle is an energy-consuming process, and studies have shown that some viruses exploit the autophagy-mediated LD degradation, termed lipophagy, for ATP production to fulfill the energy requirement and complete their infection cycle (34–36). For example, dengue virus (DENV) infection depletes LDs and TG levels in human cells. This depletion is correlated with increased autophagy and β-oxidation. Therefore, by regulating lipid metabolism, autophagy plays a role in DENV infection, which can be supplanted by the addition of exogenous FFAs (35). Here, we uncovered a similar mechanism by which WSSV hijacks shrimp autophagy for lipid utilization and ATP production. In contrast to the DENV-related finding that lipophagy facilitates the replication process, we showed that exploitation of shrimp lipophagy contributes to both the entry and replication processes in the WSSV infection cycle. In addition, we hypothesized that the monopolization of lysosomes for LD degradation might explain the result that WSSV virions escaped from lysosomal elimination. However, whether this is the case needs further clarification.

We found the functional interaction between Mindin and integrin and demonstrated that integrin inhibition blocked the role of Mindin in activating autophagy and lipid utilization and facilitating WSSV entry and replication. Based on these findings and some available reports that integrin is the receptor for Mindin from other animals (5, 11), we suppose that integrin is the receptor of Mindin in shrimp and determines its role in virus infection. Because the interaction with integrin is a prerequisite for the role of Mindin to activate autophagy and lipid utilization, the induction of autophagy by Mindin is probably through integrin. The participation of integrin in autophagy induction after receiving extracellular signals has been extensively studied. For example, osteopontin treatment in stroke mice enhances autophagy, and this enhancement can be blocked by an integrin antagonist (37). The extracellular matrix protein fibronectin can recognize the structural protein of Streptococcus and then is recruited to its receptor integrin α5β1 to activate autophagosome formation (38). Our finding that Mindin-integrin interaction leads to autophagy in a virus infection context in shrimp provides new evidence for the involvement of integrin in autophagy activation. However, currently it is unknown for the signaling events downstream of Mindin/integrin and upstream of autophagy. Clarifying the specific mechanism by which shrimp Mindin/integrin activates autophagy is of significant interest and deserves further study.

**FIG 7:**
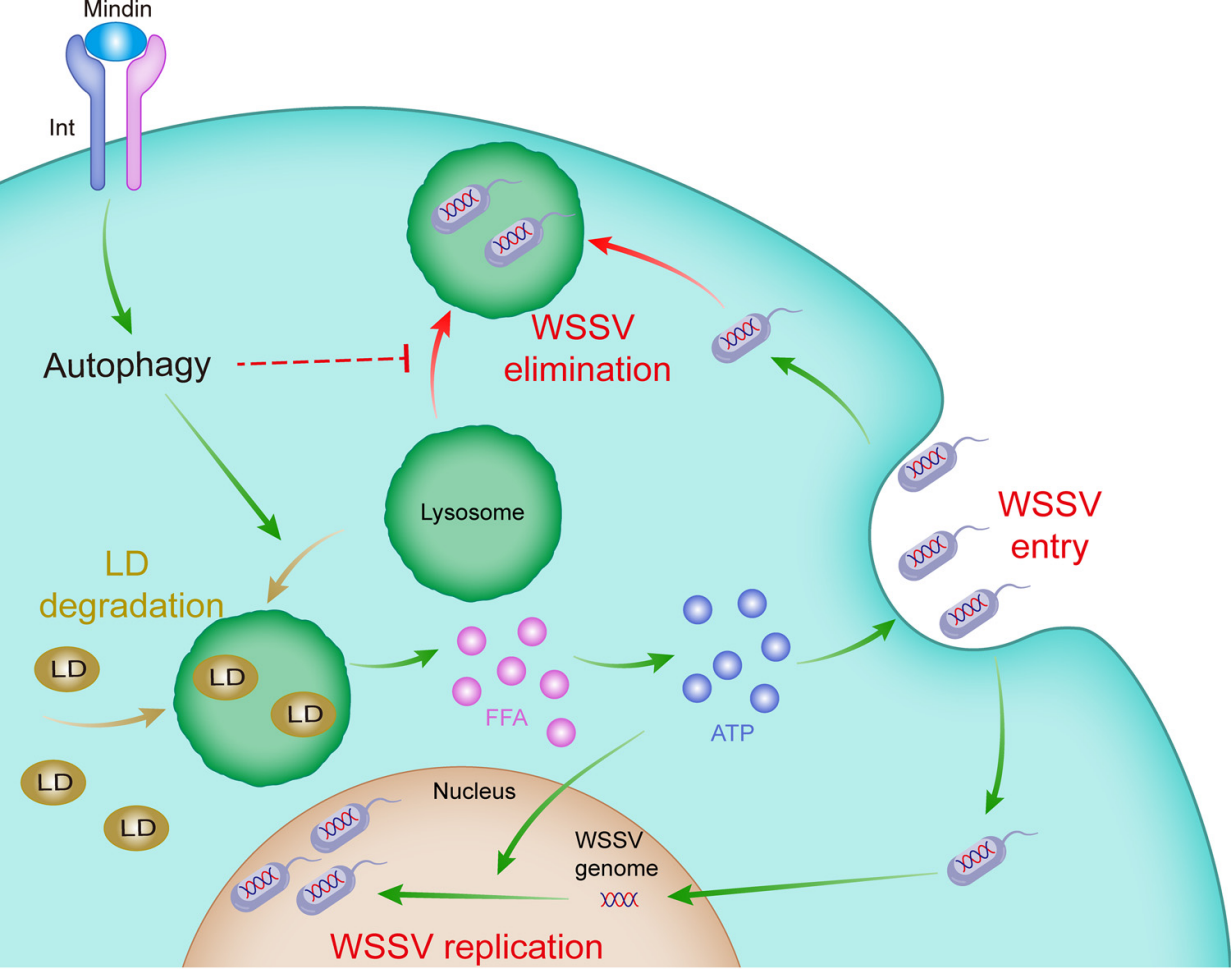
Model of the mechanism revealed in this study. Mindin interacts with integrin to induce autophagy. Autophagy regulates lipid droplet (LD) degradation to generate free fatty acids (FFAs) and produce ATP, which is essential for the entry and replication of WSSV. Moreover, the monopolization of lysosome for LD degradation may allow the virions to escape from lysosomal elimination.

In summary, we have demonstrated that the ECM protein Mindin, through integrin signaling, accelerates lipid degradation by promoting autophagy and thus facilitates the entry and replication of WSSV in shrimp (Fig. 7). This study provides new insights into the significance of Mindin in host-virus interaction and establishes a new mechanism of WSSV infection. The various nodes of this mechanism may represent new target sites to develop strategies for WSSV control in shrimp aquaculture.

## MATERIALS AND METHODS

### Animals and viral inoculum.

Healthy kuruma shrimp (body weight = 3 to 5 g) were obtained from a breeding farm in Jimo, Shandong, China. The shrimp were maintained in aerated seawater at 25°C and fed commercial diets daily in the laboratory. Shrimp were randomly selected for study. The animal-related experiments were performed with the approval of the Animal Ethical Committee of Shandong University School of Life Sciences (permit number SYDWLL-2021-98).

The original WSSV inoculum used in this study was gifted from the East China Sea Fishery Institute, Shanghai, China. To prepare successive inocula, the gills from moribund shrimp were homogenized in phosphate-buffered saline (PBS) (140 mM NaCl, 2.7 mM KCl, 10 mM Na_2_HPO_4_, 1.8 mM KH_2_PO_4_, pH 7.4) at a ratio of 1:10 (wt/vol). The homogenate was frozen and thawed twice and then centrifuged at 3,000 × *g* for 10 min at 4°C. The supernatant was filtered through a 0.45-μm filter. After determining the viral titers, the resultant filtrate was diluted to the appropriate titer using PBS and used as the WSSV inoculum. For infection assay, each shrimp was injected with WSSV inoculum at a dose of 5 × 10^5^ virus copies, with PBS injection as the control.

### Quantification of viral titers.

A recombinant plasmid harboring a WSSV *vp28* fragment was constructed according to conventional procedures. The copy number of the plasmid sample was calculated and then serially diluted. Genomic DNA, which was extracted using MagExtractor genome (Toyobo, Osaka, Japan; NPK-101) from the viral inoculum (100 μL) or tissue (10 mg), and serial plasmid samples were together analyzed using quantitative real-time PCR (qPCR). qPCR was performed using the CFX96 real-time system (Bio-Rad, Hercules, CA, USA) and the iQ SYBR green Supermix (Bio-Rad; 170-8882). The qPCR cycle conditions were 94°C for 3 min, 40 cycles of 94°C for 10 s and 60°C for 1 min, and a final dissociation protocol from 65°C to 95°C. Primers used are listed in Table S1 in the supplemental material. The standard curve generated using the data of plasmid samples was used to quantify the viral titer in the viral inoculum or infected tissues.

10.1128/mbio.02919-22.5 TABLE S1 Primers used in this study. Download Table S1, DOCX file, 0.01 MB. Copyright © 2023 Gao et al. 2023 Gao et al. https://creativecommons.org/licenses/by/4.0/ This content is distributed under the terms of the Creative Commons Attribution 4.0 International license.

### Cell lines and bacteria.

The Spodoptera frugiperda Sf9 cell line was cultured in the ESF 921 insect cell culture medium (Expression Systems, Davis, CA, USA; 96-001) containing 1.5% bovine serum (Sigma-Aldrich, Saint Louis, MO, USA; F8318), 100 IU/mL penicillin, and 100 μg/mL streptomycin sulfate at 28°C. The recombinant Escherichia coli BL21 Transetta (DE3) (TransGen Biotech, Beijing, China; CD801) strain was cultured in Luria-Bertani broth medium supplemented with 100 mg/mL of ampicillin at 37°C.

### Antibodies and inhibitors.

Polyclonal antibodies against VP28 and shrimp β-actin were generated by immunizing rabbits using the recombinant proteins and were used at 1:500 and 1:1,000 dilutions, respectively. A rabbit anti-GABARAP monoclonal antibody (Abcam, Cambridge, UK; ab109364) that can cross-react with the shrimp and *S. frugiperda* homolog was used at a 1:3,000 dilution. Horseradish peroxidase (HRP)-conjugated goat anti-rabbit secondary antibodies (Zhongshan, Beijing, China; ZB-2301) were used at a 1:2,000 dilution.

The clathrin inhibitor (chlorpromazine [CPZ]; A506232) was purchased from Sangon Biotech (Shanghai, China). The autophagy inhibitor (3-methyladenine [3-MA]; S2767), the fatty acid oxidation inhibitor (trimetazidine [TMZ]; S4543), the ATP synthase inhibitor (oligomycin A; S1478), and the integrin inhibitor (RGD peptide; S8008) were purchased from Selleck Chemicals (Houston, TX, USA). 3-MA and oligomycin A were dissolved in water containing 5% dimethyl sulfoxide (DMSO) and 30% polyethylene glycol (PEG) 300. CPZ, TMZ, and the RGD peptide were dissolved in water. CPZ was administered *in vivo* at 50 μg per animal, Oligomycin A was administered *in vivo* at 1 μg per animal, while the other inhibitors were administered at 5 μg per animal. The water-DMSO solvent was used as the control. A commercially synthesized scrambled RGD peptide (GenScript, Nanjing, China) was used as the control for the RGD peptide. The inhibitors were applied at 2 h before subsequent experiments.

### Transcriptome sequencing.

Transcriptome sequencing was performed to identify the genes regulated by WSSV. Shrimp were injected with WSSV inocula (5 × 10^5^ copies) or not. Each group consisted of 30 shrimp. The total RNAs from the gills were sampled at 24 h after infection. The commercial sequencing was performed by BGI (Shenzhen, China) using the BGISEQ 500 system with a paired-end sequencing length of 150 bp. The raw reads were filtered to obtain high-quality clean reads which were subsequently mapped to unigenes. The FPKM (fragments per kilobase per transcript per million mapped reads) value was used to determine gene expression level in different samples.

### Expression profile analysis.

RT-qPCR was performed to study the distribution and expression profiles of *Mindin* mRNAs after WSSV challenge. PCR data were analyzed using the threshold cycle (2^−ΔΔ^*^CT^*) method. *β-actin* mRNA expression was used as an internal reference. The expression level was normalized to the control group at each time point. Three independent experiments were performed, and the results represent the mean ± standard deviations (SD).

### RNAi.

A partial DNA fragment, which is specific for certain genes and linked with the T7 promoter, was used as the template for dsRNA synthesis using a T7 RNA interference (RNAi) transcription kit (Vazyme, Nanjing, China; TR102) according to the manufacturer’s instructions. The dsRNA specific for the green fluorescent protein (GFP) gene was produced similarly as a control. The dsRNAs were injected into shrimp hemocoels at a dose of 5 μg/g shrimp. RNAi efficiency was detected using RT-qPCR at 48 h after dsRNA administration.

### Western blotting.

Protein samples (100 μg/lane) were separated using 12.5% SDS-PAGE and transferred onto a nitrocellulose membrane. The membrane was blocked with 3% nonfat milk and incubated with the corresponding primary antibodies overnight at 4°C. The membrane was washed and then incubated with HRP-conjugated secondary antibodies for 2 h at room temperature. The immunoreactive signals were revealed using the High-sig ECL Western blotting substrate (Tanon, Shanghai, China; 180-5001) and captured using the Tanon 5200 chemiluminescence imaging system (Tanon). The intensities of immunoreactive protein bands were quantified using ImageJ software (NIH, Bethesda, MD, USA).

### Generation and *in vivo* application of recombinant Mindin.

The fragment corresponding to the mature peptide of Mindin was cloned into plasmid pET32a(+), which was then transformed into E. coli cells for recombinant expression under the induction of 0.2 mM isopropyl-β-d-thiogalactopyranoside. The recombinant protein was expressed as soluble protein and was purified using affinity chromatography with ProteinIso nickel-nitrilotriacetic acid (Ni-NTA) resin (TransGen Biotech; DP-101). The majority of endotoxin contamination was removed by thorough washing using cold 0.1% Triton X-114 before final elution. Purified proteins were dialyzed against PBS thoroughly. The protein concentration was determined using a Bradford protein assay kit (Sangon Biotech; C503031). The protein tags generated by the empty pET32a(+) plasmid were prepared simultaneously as controls. rMindin or the tag was injected into shrimp hemocoels with the WSSV inoculum with a dose of 5 μg per shrimp. To restore the RNAi effect, rMindin or the tag (5 μg), together with the WSSV inoculum, was injected into shrimp hemocoels at 48 h after dsRNA application.

### Survival assay.

To determine the influence of *Mindin* knockdown on shrimp mortality caused by WSSV infection, 30 shrimp were injected with the WSSV inoculum (1 × 10^6^ copies/shrimp) at 48 h after dsRNA application. To determine the effect of rMindin on virus-caused shrimp death, shrimp were injected with the WSSV inoculum together with 5 μg of rMindin or control tag. Survival rates were recorded for both groups every 12 h for 108 h.

### WSSV entry assay.

WSSV virions were labeled with FITC (Sigma-Aldrich; 1.24546). The rMindin or tag, together with FITC-WSSV (1 × 10^6^ copies/shrimp), was injected into shrimp hemocoels. The hemolymph was drawn 1 h later into cold anticoagulant (0.45 M NaCl, 10 mM KCl, 10 mM EDTA, and 10 mM HEPES, pH 7.45) and centrifuged at 800 × *g* for 10 min at 4°C to obtain hemocytes. One part of the hemocytes was resuspended in PBS and detected using flow cytometry (ImageStreamX Mark II; Merck, Kenilworth, NJ, USA). The other part was placed onto poly-l-lysine-coated glass slides and washed three times with PBS. The nuclei were labeled with 4′,6-diamidino-2-phenylindole (DAPI; AnaSpec, San Jose, CA, USA; AS-83210) for 10 min at room temperature. The slides were observed using an Olympus SpinSR10 confocal microscope (Olympus, Tokyo, Japan). The percentage of virus-positive cells was determined by counting the cells. Four visual fields were selected, and the number of cells in each visual field was not less than 100.

### Triglyceride, free fatty acid, and ATP level determination.

rMindin or control tag (5 μg) was injected into shrimp hemocoels. The hepatopancreas was collected 6 h later and divided into three parts to detect the triglyceride (TG) level, free fatty acid (FFA) level, and ATP level, using a TG content detection kit (Solarbio, Beijing, China; BC0620), an FFA content detection kit (Solarbio; BC0590), and an enhanced ATP assay kit (Beyotime, Wuhan, China; S0027), respectively, according to the manufacturer’s instructions.

### Transmission electron microscopy.

The shrimp hepatopancreas was collected at 12 h after rMindin application and incubated in 2.5% glutaraldehyde. After tissue fixation, dehydration, and resin infiltration, the tissue specimens were embedded in resin. The sample embedded in resin was transferred to an oven at 65°C for polymerization for 48 h. The resin block was then removed from the embedding model for standby application at room temperature. Then, the samples were sectioned using an ultrathin sectioning machine (Leica UC7; Leica, Wetzlar, Germany) with a section thickness of 60 to 80 nm, using a carbon-free copper mesh for section pickup. The sections on the copper mesh were stained using 2% uranyl acetate saturated alcohol solution for 8 min, washed thrice with 70% alcohol, washed thrice with ultrapure water, stained with 2.6% lead citrate solution for 8 min, avoiding carbon dioxide, washed thrice with ultrapure water, and dried with filter paper. The copper mesh sections were placed in a copper mesh box and dried at room temperature overnight. The samples were overserved under a transmission electron microscope (Fei Tecnai G2 F20; Thermo Fisher, Waltham, MA). The diameter of each lipid droplet was calculated by averaging the values quantified from different angles using ImageJ software.

### Autophagy detection.

Shrimp were injected with 5 μg of rMindin or control tag. Hemocytes were collected 6 h and 12 h later to detect the GABARAP levels using Western blotting. For the cell-related experiment, rMindin or tag (5 μg) was added to the culture medium, and the cells were collected to detect GABARAP levels 6 h and 12 h later. For the autophagosome formation detection assay, the pIEx-4-RFP-GFP-LC3-His reporter plasmid was transfected into Sf9 cells with the aid of a transfection reagent (Biodragon, Beijing, China; KX0110042). rMindin or tag (5 μg) was added to the culture medium 48 h later. The fluorescence was detected another 12 h later using the Olympus SpinSR10 confocal microscope. To quantify cell fluorescence intensity, a line was drawn across the cell of interest and the line-scan graphs were generated to determine the intensity value of the pixels by using ImageJ software.

### Lysosomal staining.

Shrimp hemocytes were collected at 3 h after rMindin and virions injection and processed with 4% paraformaldehyde for 10 min. The hemocytes were placed onto poly-l-lysine-coated glass slides. Lysosomes were stained using a lysosome staining kit (Sangon Biotech; E607506) according to the manufacturer’s instructions. The slides were observed using the Olympus SpinSR10 confocal microscope. ImageJ software was used to determine the colocalization of the virus and lysosome. The colocalization rate was quantified as (WSSV colocalized with lysosomes/WSSV in hemocytes) × 100%.

### Statistical analysis.

The images shown in this study are representative of three independent experiments, and all bar charts show the mean ± SD derived from three or four independent repeats. At least five shrimp were used for each sample. Two-tailed Student’s *t* test or one-way analysis of variance (ANOVA) was used to calculate the statistical significance using GraphPad Prism 8 (GraphPad Inc., La Jolla, CA, USA), and a significant difference was accepted at a *P* value of <0.05. The log rank (Mantel-Cox) test in GraphPad Prism 8 was used to analyze the survival assay data.

### Data availability.

All data are included in the article and in the supplemental material.
