# Supplementary figures and images for: Mindin Activates Autophagy for Lipid Utilization and Facilitates White Spot Syndrome Virus Infection in Shrimp
Source: mBio. 2023 Feb 13;14(2):e02919-22. doi: 10.1128/mbio.02919-22 (PMC10127999; doi:10.1128/mbio.02919-22)

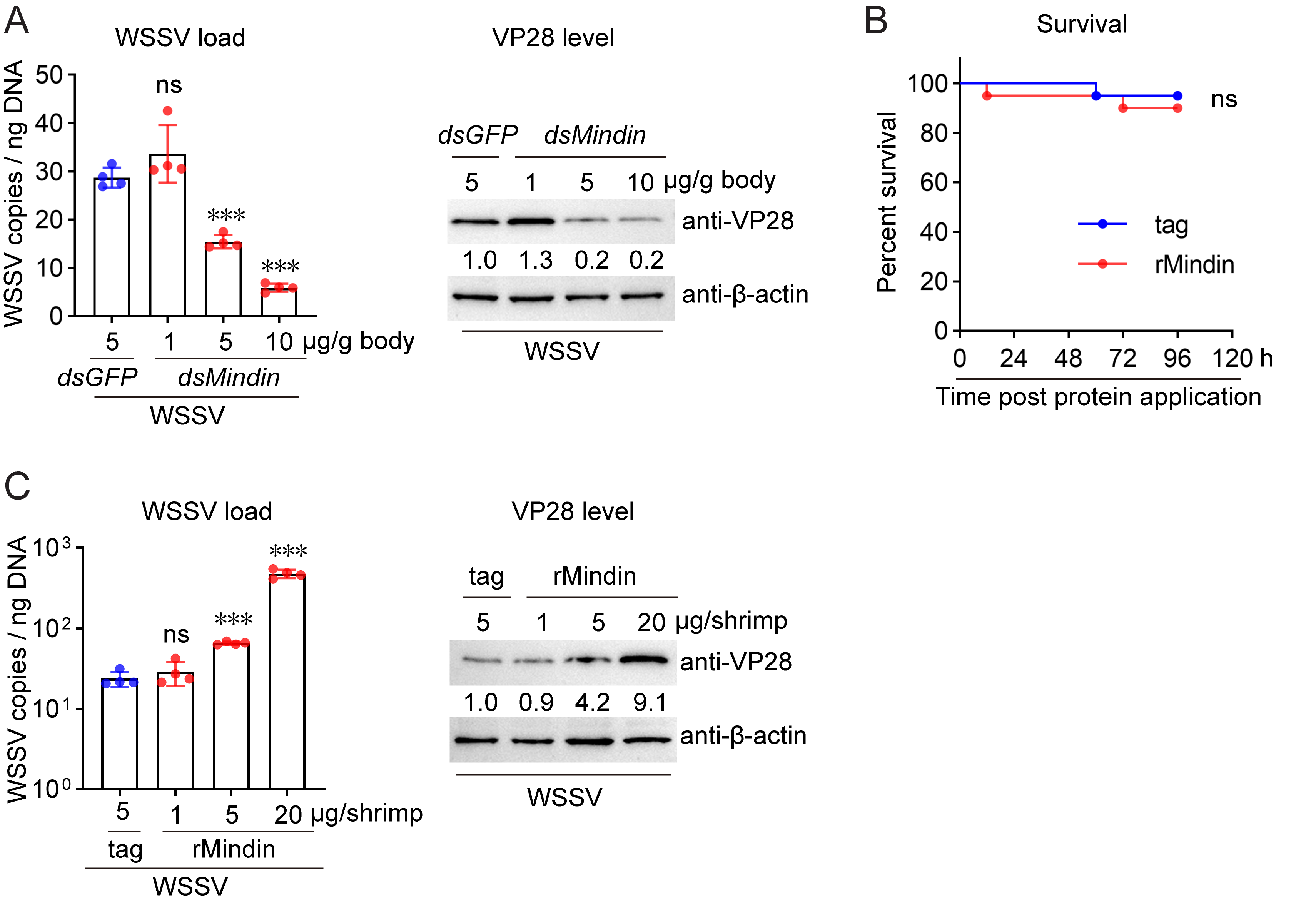

Supplement: FIG S1 [file mbio.02919-22-s0001.tif]

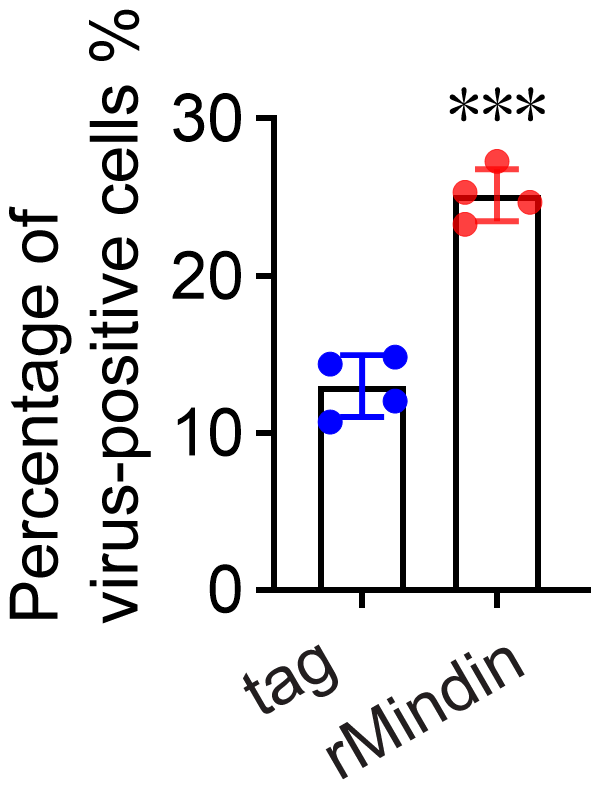

Supplement: FIG S2 [file mbio.02919-22-s0002.tif]

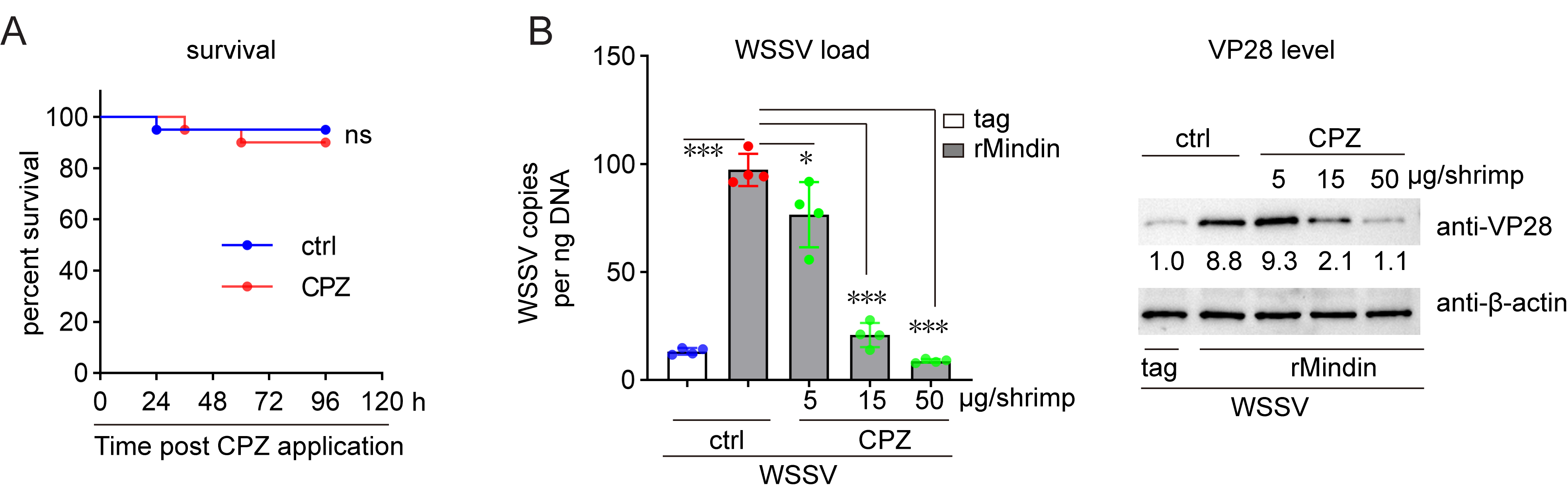

Supplement: FIG S3 [file mbio.02919-22-s0003.tif]

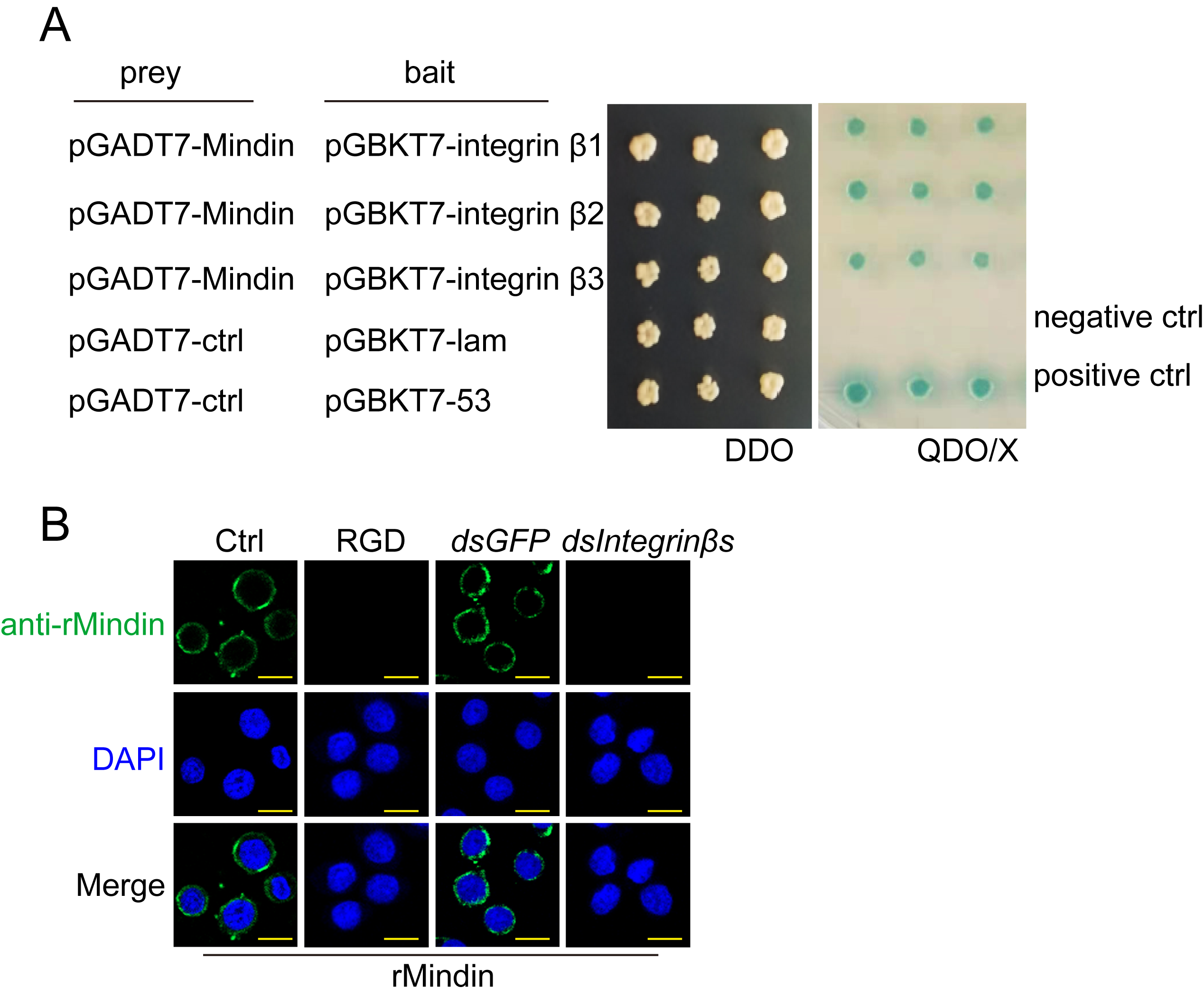

Supplement: FIG S4 [file mbio.02919-22-s0004.tif]
