# Supplementary material for: Mindin Activates Autophagy for Lipid Utilization and Facilitates White Spot Syndrome Virus Infection in Shrimp
Source: mBio. 2023 Feb 13;14(2):e02919-22. doi: 10.1128/mbio.02919-22 (PMC10127999; doi:10.1128/mbio.02919-22)
Supplement: TABLE S1 [file mbio.02919-22-s0005.docx]

| Primers | Sequence (5’-3’) |
| --- | --- |
| **(q)RT-PCR** |  |
| β-actinRTF | CAGCCTTCCTTCCTGGGTATGG |
| β-actinRTR | GAGGGAGCGAGGGCAGTGATT |
| VP28RTF | AGCTCCAACACCTCCTCCTTCA |
| VP28RTR | TTACTCGGTCTCAGTGCCAGA |
| MindinRTF | GGCACAGACAACGGCTTCA |
| MindinRTR | TTACTCACTTCCTGGTCACGGT |
| ClathrinRTF | ATTTGATAGAGTTTCGTCG |
| ClathrinRTR | GCAGGTCGTAGCAGTGGA |
| Integrinβ1RTF | CCCGTCGTCTTTTGGTCT |
| Integrinβ1RTR | TGGAGGAATTGGCAGTGAG |
| Integrinβ2RTF | ACGAGCCTTTGAGTGATGTCT |
| Integrinβ2RTR | CGACGAAAGAACCGAAACC |
| Integrinβ3RTF | TTCATCGGAAACTGGTGC |
| Integrinβ3RTR | ATCTGAGGGCTTGGTAGCT |
| **RNAi** |  |
| MindiniF | GCGTAATACGACTCACTATAGGGATCCCTGCTACCACGCCTACT |
| MindiniR | GCGTAATACGACTCACTATAGGGCCAAAATGCTTTACCGATACTCC |
| Integrinβ1iF | GCGTAATACGACTCACTATAGGGAATGTATCCAGACACCCAA |
| Integrinβ1iR | GCGTAATACGACTCACTATAGGAAAGGAACCAAAGCCAAG |
| Integrinβ2iF | GCGTAATACGACTCACTATAGGGACCCAAGACCAGAGTGAG |
| Integrinβ2iR | GCGTAATACGACTCACTATAGGGACGAAAGAACCGAAACC |
| Integrinβ3iF | GCGTAATACGACTCACTATAGGCCGCATTATTGGTGGTCT |
| Integrinβ3iR | GCGTAATACGACTCACTATAGGGGCAACACTGGCTTATCG |
| ClathriniF | GCGTAATACGACTCACTATAGGGCCCAACGCTGGTTATGCT |
| ClathriniR | GCGTAATACGACTCACTATAGGGTGACCTGACCGCCTCTAC |
| **Recombinant expression** |  |
| MindinEF | CCGGAATTCACCTGTGACCCCAACAAACTG |
| MindinER | AAATATGCGGCCGCCTACCAGTCGAAGTACTTGT |
